# Exploring the genetic makeup of *Xanthomonas* species causing bacterial spot in Taiwan: evidence of population shift and local adaptation

**DOI:** 10.3389/fmicb.2024.1408885

**Published:** 2024-05-23

**Authors:** Jaw-Rong Chen, Kevin Aguirre-Carvajal, Dao-Yuan Xue, Hung-Chia Chang, Lourena Arone-Maxwell, Ya-Ping Lin, Vinicio Armijos-Jaramillo, Ricardo Oliva

**Affiliations:** ^1^Safe and Sustainable Value Chain, World Vegetable Center, Shanhua, Taiwan; ^2^Research Center of Information and Communication Technologies, University of A Coruña, A Coruña, Spain; ^3^Bio-Cheminformatics Research Group, Universidad de Las Américas, Quito, Ecuador; ^4^Seed and Seedling Management Section, Taiwan Seed Improvement and Propagation Station, Ministry of Agriculture, Taichung, Taiwan; ^5^Facultad de Ingeniería y Ciencias Aplicadas, Universidad de Las Américas, Quito, Ecuador

**Keywords:** bacterial spot, local adpatation, diversification, pathogen introduction, effectors

## Abstract

The introduction of plant pathogens can quickly reshape disease dynamics in island agro-ecologies, representing a continuous challenge for local crop management strategies. *Xanthomonas* pathogens causing tomato bacterial spot were probably introduced in Taiwan several decades ago, creating a unique opportunity to study the genetic makeup and adaptive response of this alien population. We examined the phenotypic and genotypic identity of 669 pathogen entries collected across different regions of Taiwan in the last three decades. The analysis detected a major population shift, where *X. euvesicatoria* and *X. vesicatoria* races T1 and T2 were replaced by new races of *X. perforans*. After its introduction, race T4 quickly became dominant in all tomato-growing areas of the island. The genomic analysis of 317 global genomes indicates that the *Xanthomonas* population in Taiwan has a narrow genetic background, most likely resulting from a small number of colonization events. However, despite the apparent genetic uniformity, *X. perforans* race T4 shows multiple phenotypic responses in tomato lines. Additionally, an in-depth analysis of effector composition suggests diversification in response to local adaptation. These include unique mutations on *avrXv3* which might allow the pathogen to overcome *Xv3/Rx4* resistance gene. The findings underscore the dynamic evolution of a pathogen when introduced in a semi-isolated environment and provide insights into the potential management strategies for this important disease of tomato.

## Introduction

Tomato (*Solanum lycopersicum* L.) is one of the most important vegetable crops worldwide. Yet, most tomato-growing areas are challenged by bacterial spot caused by *Xanthomona*s species. This disease induces necrotic spots on aboveground tissues, leading to defoliation, yield losses, and reduced fruit quality and marketability (Pohronezny and Volin, 1983). An increase in the incidence of bacterial spot has been reported in different regions of the world (Hamza et al., 2010; Horvath et al., 2012; Beran et al., 2014; Kebede et al., 2014; Abbasi et al., 2015; Potnis et al., 2015; Osdaghi et al., 2016; Araújo et al., 2017; Burlakoti et al., 2018). In the field, however, symptoms can be caused by different *Xanthomonas* species, including *X. vesicatoria*, *X. gardneri*, *X. euvesicatoria*, and, *X. perforans* (Jones et al., 2004). A more recent classification also suggests that *X. euvesicatoria* may include two distinct pathovars: *euvesicatoira* and *perforans* (Constantin et al., 2016). While historically, *X. euvesicatoria* and *X. vesicatoria* have been the predominant species infecting tomato, recent reports pointed out the emergence of *X. perforans* in the United States (Horvath et al., 2012; Ma, 2015), Ethiopia (Kebede et al., 2014), Canada (Abbasi et al., 2015), and Brazil (Araújo et al., 2017), among others. *Xanthomonas* pathogens causing bacterial spot can be classified into at least five different races (Asuta-Monge et al., 2000; Jones et al., 2004). Rapid changes in the pathogen population represent a significant challenge for disease management strategies because no tomato variates show resistance to all of these races. While the disease is well-studied, the emergence of new *Xanthomonas* strains and races poses a continuous challenge for disease management. Understanding the genetic makeup and adaptation pathways of these bacterial pathogens is crucial for developing effective control strategies.

To proliferate in the plant tissues, most bacteria pathogens rely on the translocation of type III effector proteins into the host cell. The *Xanthomonas* outer proteins (Xop) are a big group of effectors with diverse biochemical functions that target different subcellular compartments (Büttner and Bonas, 2010). Effectors interfere with plant processes to modulate plant defense response and facilitate access to host nutrients (Toruño et al., 2016). During host colonization, *Xanthomonas* are known to deliver between 23 and 37 different type III effectors, although individual strains might carry a variable repertoire, most likely due to redundancy in functions (Büttner and Bonas, 2010). Plants also evolved networks of protein sensors that recognize the effectors and elicit a response that restricts pathogen advance (Jones and Dangl, 2006). For instance, the tomato resistance protein Bs4 triggers a hypersensitive response (HR) when recognizing the cognate avirulence protein AvrBs4 from *Xanthomonas* T1 race (Ballvora et al., 2001). The same is true for the effector AvrXv3 (xopAF) from *Xanthomonas* T3 race, which is recognized by the nucleotide-binding leucine-rich repeat (NBS-LRR) protein Xv3/Rx4, present in *S. pimpinellifolium* (Zhang et al., 2021). Understanding the distribution of effector repertoires in pathogen populations is crucial for supporting disease management strategies and guiding breeding efforts in modern agriculture.

Plant pathogens often experience a process of selection and diversification as they adapt to new hosts or environments (Brasier, 1987). This is particularly true in semi-isolated areas, such as islands, where introducing new pathogens can drastically shape the existing population structure. The study of *Xanthomonas* species in Taiwan is particularly relevant since tomato cultivation is relatively new. While the Dutch introduced tomatoes as an ornamental plant in 1622 (Chen, 2005), high-scale cultivation only started when varieties like Ponderosa and Marglobe were brought in from the United States in the early 1930s. Since then, tomato production has occupied the central and southern areas of the island, where hot and humid environments are conducive to the disease. The first report of bacterial spot was in the early 1930s (Okabe, 1933), and recent reports suggest the presence of *X. vesicatoria*, *X. euvesicatoria*, and *X. perforans* (Lue et al., 2010; Burlakoti et al., 2018). This study aims to understand the genetic makeup of *Xanthomonas* pathogens causing bacterial spot in Taiwan and uncover the pathway of adaptation to the novel environment.

## Materials and methods

### Pathogen collection and molecular characterization

We analyzed the records of *Xanthomonas* strains causing bacterial spots in Taiwan over the past 34 years (Supplementary Table S1). Of these records, 293 were obtained from a previous study conducted by Burlakoti et al. (2018), which included collections from 1989 to 2016. We also included 376 bacterial strains isolated from tomato plants displaying disease symptoms between 2017 and 2023 (Supplementary Table S1). All bacterial strains were stored at −80°C in 30% glycerol at the World Vegetable Center. DNA was extracted through the boiled bacterial suspension (concentration: 1*10^8^ CFU/mL) for each bacterial culture. The species identification was done using touchdown multiplex-PCR with species-specific primers following the reaction described in Lue et al. (2010). The primer set of species-specific primers for *X. euvesicatoria* (RST13/RST14), *X. vesicatoria* (Xv-1f/Xv-1r), *X. perforans* (HpaF-f/HpaF-r), *X. gardneri* (BS-XgF/BS-XgR). The PCR products were confirmed using 1.5% agarose gel electrophoresis. The gel was stained with HealthView Nucleic Acid Stain (Cat. GN-NAS-100) and visualized under ultraviolet (UV) light. *X. euvesicatoria*, *X. vesicatoria*, *X. perforans*, and *X. gardneri* amplified a product of 560-bp, 197-bp, 300-bp, and 154-bp, respectively.

### Race identification, pathogenicity test, and resistance screening

All strains were characterized into Races using the hypersensitive reactions on 4 differential hosts (Stall et al., 2009), including *S. lycopersicum* Bonny best L305 (without resistance gene), Hawaii7998 (with resistance gene *rx-1*, *rx-2*, *rx-3*), Hawaii7981 (with resistance gene *Xv3*), and *S. pennelii* LA716 (with resistance gene *Xv4*). Strains were classified into T0, T1, T2, T3, T4, and T5 Races. Furthermore, 1 T5, 24 T4, and 5 T3 *X. perforans* strains were selected to characterize additional virulence patterns. The strains were inoculated on *S. lycopersicum* L305 (susceptible line) and H7981 (with resistance gene *Xv3*), *S. pimpinellifolium* L3707 (resistant to race T3 and T4), and *S. lycopersicum* var. *cerasiforme* PI114490 (high-level resistance) using the dip-inoculation method. Seedlings at the 4–6 true leaves stage were inverted and immersed in bacterial suspension (1*10^8^ CFU/mL) containing 0.03% surfactant (Silwett L^−77^) for 30 s. The inoculated plants were kept in a growth room with warm and high humidity conditions (first 48 h at 90 ± 5% RH, then transfer to 80 ± 5% RH and 28–35°C), suitable for disease progress. The experiment was laid out in a randomized complete block design (RCBD) with 3 replications, each replication containing 5 plants/strain/host genotypes. Disease severity was recorded according to Horsfall-Barratt’s rating scale (0–11) at 14 days post-inoculation, where 0 = 0%, 1 = 0–3%, 2 = 3–6%, 3 = 6–12%, 4 = 12–25%, 5 = 25–50%, 6 = 50–75%, 7 = 75–88%, 8 = 88–94%, 9 = 94–97%, 10 = 97–100%, 11 = 100% diseased tissue (Horsfall and Barratt, 1945). The disease reaction of tomato genotypes was categorized as referred by Hutton et al. (2010), where resistance (R) = mean scale ≤4; moderate resistance (M) = 4 < mean scale ≤5; susceptible (S) = mean scale >5. These patterns allowed the selection of a representative strain from the main virulence group for resistance screening. A total of 86 *S. pimpinellifolium* and 14 *S. lycopersicum* var. *cerasiforme* accessions were screened with a representative strain to identify new sources of resistance. The resistance screening method was the same as described above.

### Genome assembly, core genome determination, and spanning tree reconstruction

The genomes were sequenced at BGI Genomics in China, using a DNBseq platform with a short-insert library to obtain 150 bp paired end reads. The Velvet algorithm was utilized for assembling the *Xanthomonas* genomes (Zerbino and Birney, 2008). To determine the optimal K-mer size and coverage cutoff for each specific genome, the VelvetOptimiser script was employed (Gladman et al., 2017). For detailed statistics of the genome sequencing and assembly, refer to Supplementary Table S3. Subsequently, the contigs originating from *X. euvesicatoria* and *X. perforans* strains were mapped using the genomic reference of *X. euvesicatoria* pv. vesicatoria str. 85–10 (NC_007508) and *X. perforans* strain GEV872 (NZ_CP116305) respectively. Correspondingly, the contigs derived from *X. vesicatoria* were aligned against the genomic reference of *X. vesicatoria* strain LMG911 (NZ_CP018725.1) using the Geneious plugin minimap2 (Li, 2018). This approach yielded 95.5% (on average) of the reference genome for both Taiwanese *X. vesicatoria* and *X. perforans* genomes and 94.4% in average for *X. euvesicatoria* genomes. Genome annotation was done within Geneious version 2023.2.1.^1^ We also annotated the genomes contained within the scaffolds generated by Velvet. The core genome of *X. euvesicatoria*, *X. vesicatoria*, and *X. perforans* was leveraged with all the available annotated genomes in GenBank (as of December 22, 2022), along with additional strains from Taiwan. The get_homologues software (Contreras-Moreira et al., 2017) was applied to discern the core genomic elements. Subsequently, the identified core genome was input for selecting phylogenomic markers using the get_phylomarkers software (Vinuesa et al., 2018). The phylogenetic tree was built with the 62 robust markers as ascertained by the get_phylomarkers approach. Spanning trees were constructed using a dataset of 296 genomes available in the NCBI database for *X. perforans*, *X. euvesicatoria*, and *X. vesicatoria* in conjunction with the 21 Taiwan assembled genomes. Thirty informative phylomarkers were selected and concatenated to form a representative sequence for each genome. Software PHYLOViZ (Francisco et al., 2012) was used to reconstruct the spanning tree.

### Effectors annotation and analysis

To establish a dataset of *Xanthomonas* effectors, we used the database resource contained in the Euro Xanth webpage.^2^ This resource compiles 80 effectors, out of which 70 possess reference sequences in the NCBI Nucleotide database. The corresponding effector genes were obtained in GenBank format. With the objective of discerning effectors present in the Taiwanese genomes, the Geneious annotation tool was employed. Annotation of all the 70 reference sequences was conducted using a 70% similarity threshold. As a result, 48 effectors were identified among the Taiwanese genomes. These effectors include *avrBs1*, *avrBs2*, *avrXccA1*, *HpaA*, *xopA*, *xopAA*, *xopAD*, *xopAE*, *xopAF*, *xopAG1*, *xopAI*, *xopAJ*, *xopAK*, *xopAM*, *xopAP*, *xopAQ*, *xopAR*, *xopAU*, *xopAV1*, *xopAW*, *xopAX*, *xopAY*, *xopAZ*, *xopB*, *xopBA*, *xopC*, *xopD*, *xopE*, *xopE2*, *xopE3*, *xopF1*, *xopF2*, *xopG*, *xopI*, *xopJ1*, *xopJ2*, *xopJ3*, *xopJ4*, *xopK*, *xopM*, *xopN*, *xopO*, *xopP*, *xopQ*, *xopS*, *xopV*, *xopX*, and *xopZ1*. The sequences of the identified effectors in each genome were subsequently extracted, translated, and grouped. A BLASTp analysis was conducted using the GenBank database, focusing on the species *X. perforans*, *X. euvesicatoria*, and *X. vesicatoria*. BLASTp results that match with the effector annotation were retained. We identified unique alleles by performing protein alignments and all the BLAST hits found in the databases.

## Results and discussion

### *Xanthomonas* populations in Taiwan experienced a major shift

**Figure 1:**
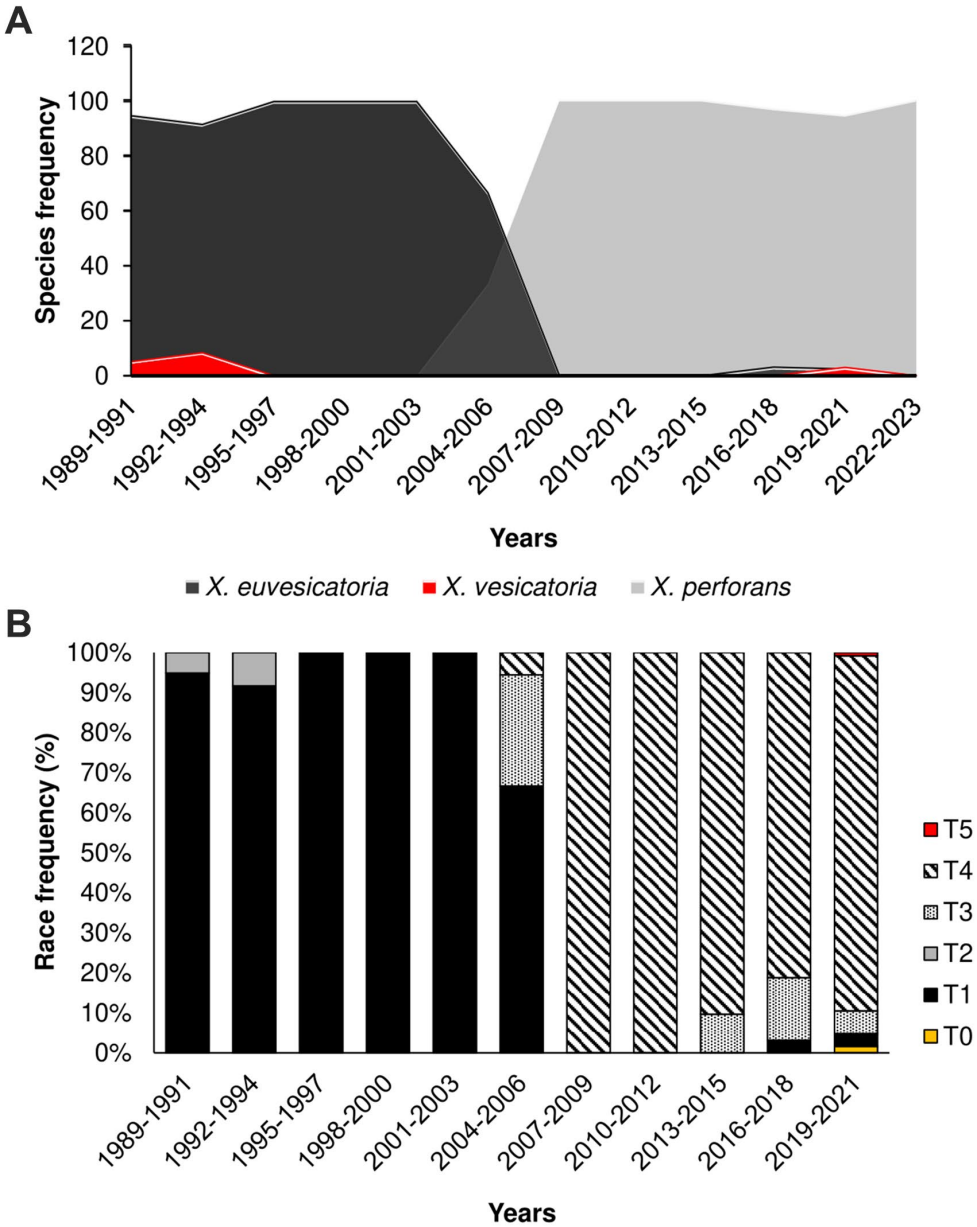
The population shift of *Xanthomonas* species causing Bacterial Spot in Taiwan started in 2004–2006; **(A)** Relative frequency of *Xanthomonas* species in the last 34 years (1989–2023); **(B)** Relative frequency of race groups among *Xanthomonas* species causing bacteria spot collected in Taiwan.

To investigate the historical dynamics of *Xanthomonas* populations, we mined a dataset of 669 strains causing bacterial spot in Taiwan (Supplementary Table S1). The strains were isolated across 82 tomato-growing areas in 13 major districts in the last 34 years (1989–2023). Along with previous samples collected between 1989 and 2016 (293), we also obtained 376 *Xanthomonas* strains during the 2017 to 2023 surveys. Overall, we identified three main *Xanthomonas* species in our collection using DNA markers: *X. vesicatoria*, *X. euvesicatoria*, and *X. perforans* (Figure 1; Supplementary Table S1). We were unable to find any samples corresponding to *X. gardneri*. We plotted the time distribution of all *Xanthomonas* strains and found a significant shift in *Xanthomonas* species, a trend that began in the early 2000s, as previously described in Burlakoti et al. (2018), and persists to the present day. The data consistently show the replacement of *X. euvesicatoria* and *X. vesicatoria* by *X. perforans* as the dominant population from the early 2000s until 2023 (Figure 1A).

Changes in *Xanthomonas* species prevalence have also been observed in different regions over the past two decades (Kebede et al., 2014; Timilsina et al., 2015; Araújo et al., 2017). For instance, Timilsina et al. (2019) reported a shift from *X. euvesicatoria* to *X. perforans* in Florida’s tomato-growing areas. Other reports describe a similar phenomenon in North Carolina, Indiana, Ohio, and other regions in the Midwest United States (Ma et al., 2011; Horvath et al., 2012; Egel et al., 2018; Adhikari et al., 2019; Bernal et al., 2022). Rapid shifts in pathogen species of *Xanthomonas* are likely the result of fitness advantage. For instance, resistance to copper-based compounds, commonly used in Taiwan, might explain how resistance populations might become dominant in time. However, Burlakoti et al. (2018) find no significant differences in copper resistance phenotypes among *Xanthomonas* species. It has been suggested that the increase in *X. perforans* may be due to the production of antimicrobial peptides, also known as bacteriocins (Marutani-Hert et al., 2020). However, this factor does not fully explain why pepper does not experience a similar surge in *X. perforans* infections in Taiwan (Burlakoti et al., 2018). It is more plausible that other factors, such as pathogenicity and resistance components, play a pivotal role in shaping the observed trends of bacterial spot in tomato and pepper (Adhikari et al., 2020). Further investigation into these factors is essential to comprehensively understand the dynamics of *Xanthomonas* species shifts in different crops and regions.

### *Xanthomonas perforans* T4 Race is now dominant in Taiwan

To investigate the change in pathogenicity of the *Xanthomonas* population in Taiwan, we inoculated 156 strains from the late collection onto four tomato differentials. All the races (T0-T5) were identified. The integrated data on pathogenicity unveiled a shift from T1 and T2 races, collected before the 2000s, toward T3 and T4 races collected after this period (Figure 1B). Race T4 showed remarkable prevalence, with more than 95% of the samples collected after 2015 (Figure 1B), suggesting a fitness advantage of this pathogenicity group over T3. Race designation as T3 or T4 is based only on the presence or absence of hypersensitive response (HR) caused by the recognition of avrXv3 by the cognate Xv3 (Robbins et al., 2009). Further genetic analysis found no differences between T3 and T4 genomes, except for crucial mutations that disrupt the *avrXv3* effector in T4 strains (details below). In Taiwan, there is no report on the frequency of *Xv3*, but a draft estimate suggests that 20% of tomato hybrids might have *Xv3* (Chou, personal communication). As a result, one possible explanation for the increase in T4 races is that the host is driving selection due to the presence of *Xv3*. However, a recent population swift in the Midwestern United States involving an *X. perforans* T4 population occurred in the absence of the resistance *Xv3* locus (Bernal et al., 2022), suggesting that other factors might also be involved. This new T4 population (2017–2020) appears to be genetically identical, probably due to recent migration events (Bernal et al., 2022).

**Figure 2:**
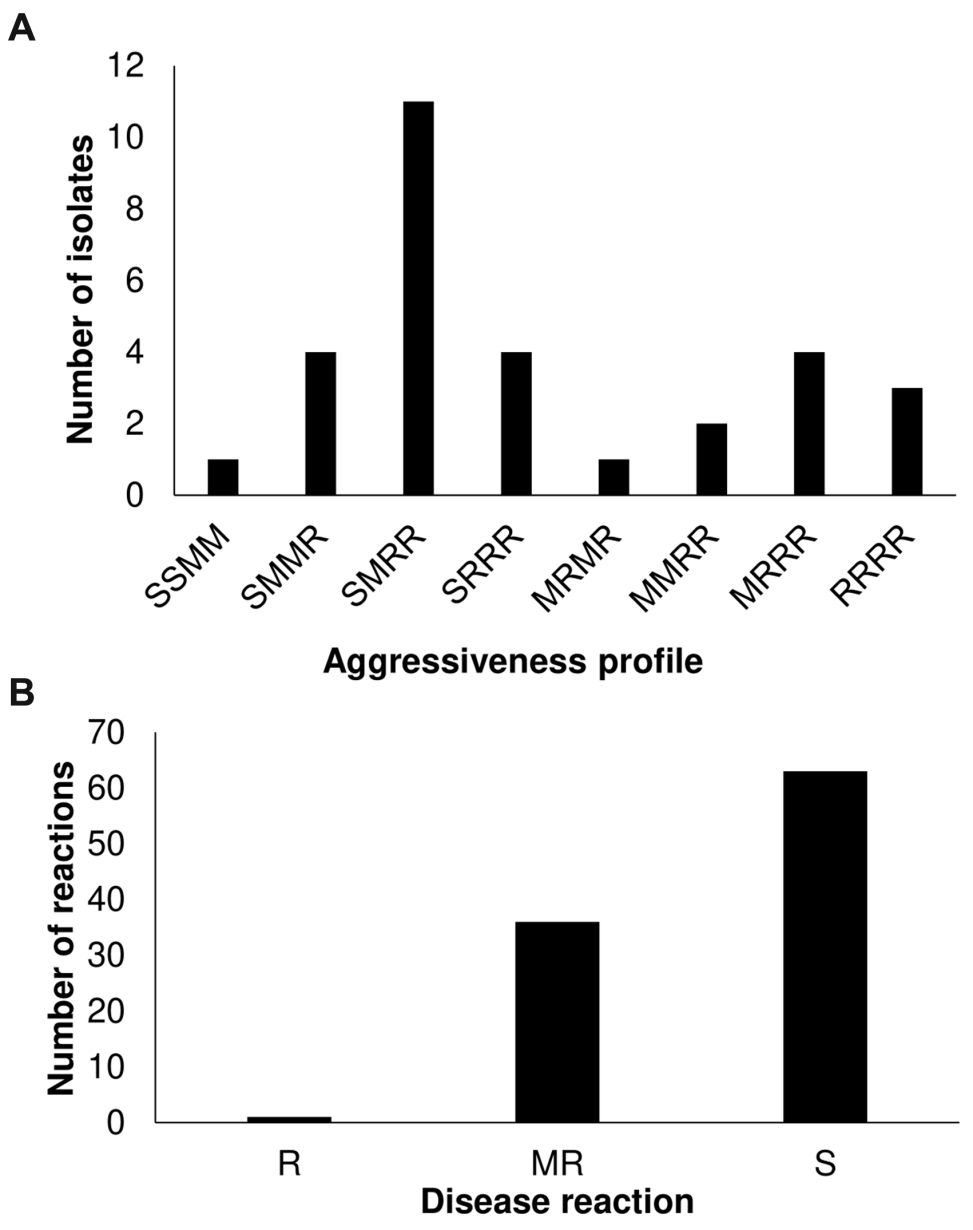
Characterization of *Xanthomonas perforans* race T3 and T4 strains on Solanaceae germplasm. **(A)** Virulence patterns of 30 *X. perforans* race T3, T4, and T5 against four reference tomato accessions. The letters in order represent the disease reaction of tomato variety (L305, H7981, L3707, PI114490) classified as resistance (R): mean scale <=4, moderate resistance (M): 4 < mean scale <=5, and susceptible (S): mean scale >5. **(B)** Screening of 86 *S. pimpinellifolium* and 14 *S. lycopersicum* var. *cerasiforme* accessions with strain Xant314.

We further identified additional virulence patterns within T3, T4, and T5 populations by inoculating 1 T5, 24 T4, and 5 T3 strains on a different tomato panel. Based on the phenotypic response, which was classified as resistance (R), moderate resistance (M), and susceptible (S), we found eight different pattern combinations among the small sample (Figure 2A; Supplementary Figure S1; Supplementary Table S1). Race T4 strains were classified into 8 patterns, suggesting that the *X. perforans* population in Taiwan harbors diverse phenotypes and might be going through a process of local diversification. Phenotypic heterogeneity in plant pathogens in response to the environment has been described even for clonal populations (Delgado et al., 2013; Mariz-Ponte et al., 2022). Finally, we look for new sources of resistance against predominant virulent disease by screening the highly virulent T4 strain (Xant314) against an *S. pimpinellifolium*-diversity panel. We found only one accession (VI007032) with high resistance levels (Figure 2B; Supplementary Table S2). VI007032, which showed a slight symptom of bacterial spot, will be further tested as a potential resistance donor. The data suggest a fitness advantage of race T4 but also a process of phenotypic diversification.

### *Xanthomonas* in Taiwan come from a narrow genetic background

**Figure 3:**
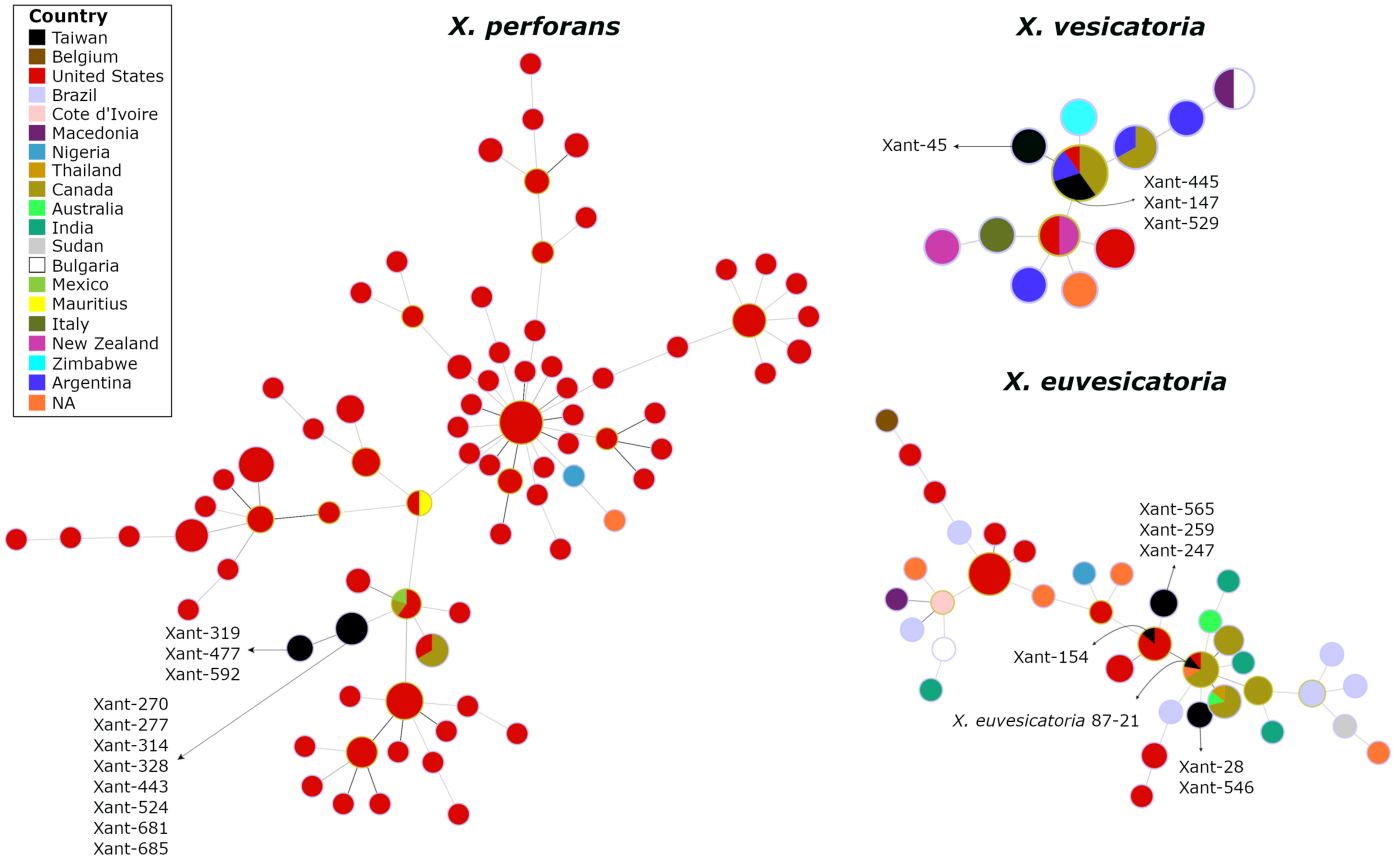
The spanning tree of *Xanthomonas* species causing Bacterial Spot in Taiwan depicts the potential linkage of *X. perforans*, *X, euvesicatoria*, and *X. vesicatoria* to North American populations. The circles represent genetic clusters that are linked by lines. The colors inside the circles represent the countries where those clusters have been found. The genomes from Taiwan are depicted in black.

To investigate the genetic variation of *Xanthomonas* causing bacterial spot in Taiwan, we obtained complete genome sequences for 21 representative strains collected in highland and lowland tomato-growing areas over 30 years (Supplementary Table S2). The assembled genomes (235-245X coverage range) represent *X. perforans* (11), *X. euvesicatoria* (6), *X. vesicatoria* (4), and include T1 to T5 races (Supplementary Table S3). Overall, we found indications of a narrow genetic background within *Xanthomonas* species from Taiwan. Using phylogenetic reconstruction from 317 genomes, we clearly distinguish between *Xanthomonas* strains clustering at the species level and forming single clusters across the tree (Supplementary Figure S2). The spanning tree constructed from concatenated phylomarkers linked the Taiwanese *X. perforans* strains to a single North American genotype, particularly represented in the United States and Canada (Figure 3). We identified two clades that derived from the same *X. perforans* genotypes. The first clade included strains collected in 2005, and the second clade included strains from 2016 (Figure 3).

Interestingly, we found no genetic substructure that clusters T3, T4, or T5 groups, suggesting that these might be derived. In addition, the Split decomposition tree found no evidence of recombination among *Xanthomonas* in Taiwan (data not shown). These preliminary results challenge the hypothesis of multiple colonization events on the island and suggest that *X. perforans* in Taiwan result from a single introduction (one or few genotypes). It is worth noting that the abundance of genomes from North America in our databases may introduce some bias into the results. The lack of recombination, the absence of structure among races (Supplementary Table S1), and the virulence pattern on reference tomato accessions (Figure 2) also suggest that the original *X. perforans* diversified locally in the last two decades. A similar scenario has been described recently where a clonal population of *X. perforans* that dominate extensive areas shows the accumulation of genetic variation (Bernal et al., 2022). The case of *X. vesicatoria* appears to follow the trend, as it is characterized by a single genotype. A single introduction, probably during the late 80s, was likely responsible for bringing a race T1 to Taiwan (Figure 3). The spanning tree of *X. euvesicatoria* detected two main clusters, suggesting more than one colonization event. Both colonization events of race T2 are likely to come from North America (Figure 3). One cluster was associated with Taiwanese strain 87–21, collected in the late 80s (Bouzar et al., 1994), and the other was associated with strain Xant154, collected in 1994 (Burlakoti et al., 2018). The genetic data of representative *Xanthomonas* genomes from Taiwan points to North America as the main source. Discrete genotypes were likely introduced and then diversified locally. The pathogen could have been introduced during the importation of tomato varieties, but other explanations cannot be excluded.

### The effector composition of *Xanthomonas* populations reveals diversification and local adaptation

**Figure 4:**
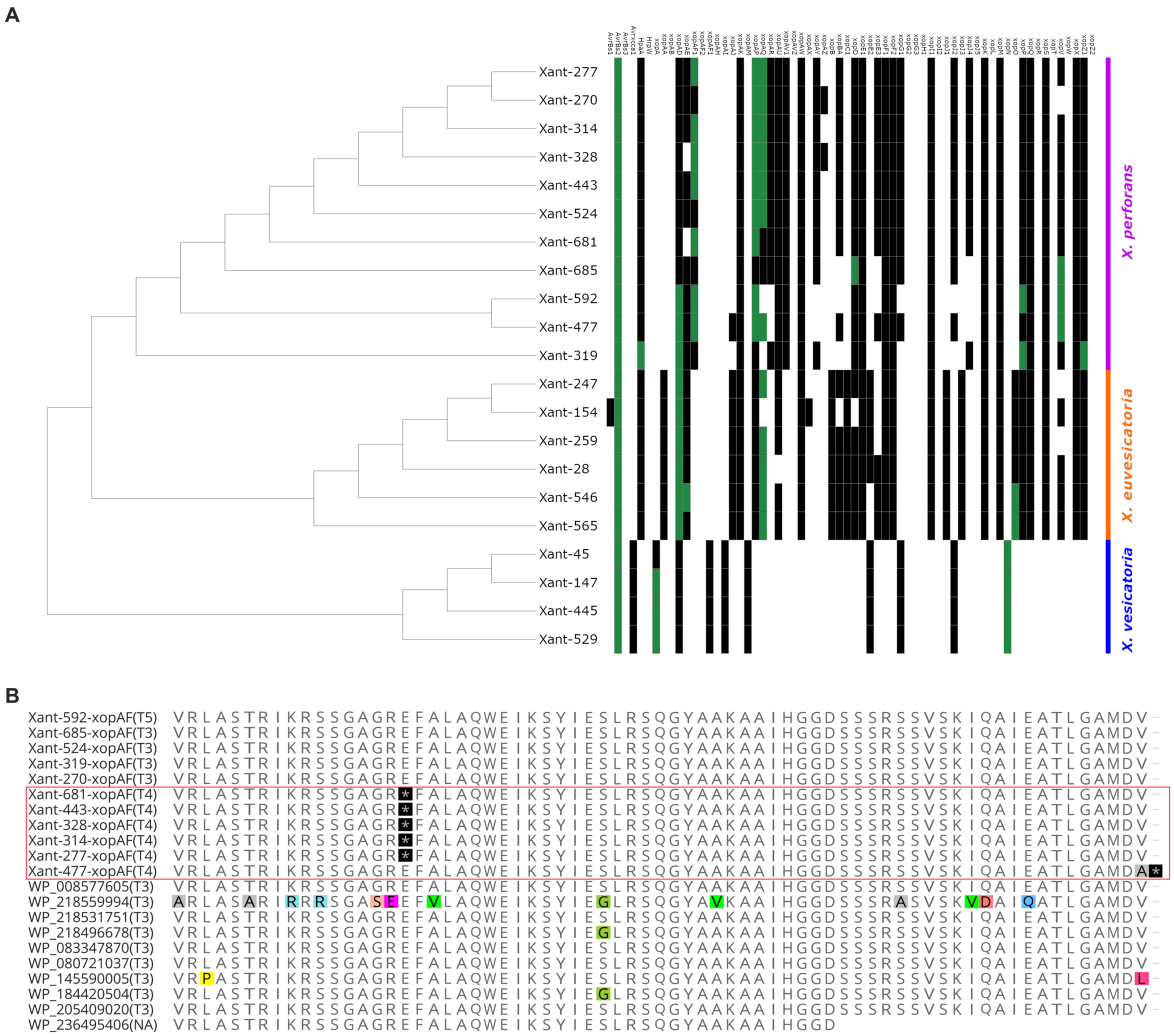
Evidence of distinctive effector composition in *Xanthomonas* from Taiwan. **(A)** The heat map tree illustrates the presence or absence of *Xanthomonas* outer proteins (Xop) present in each strain. Green squares indicate the presence of a unique allele found in a *Xanthomonas* genome. **(B)** Partial sequence alignment of XopAF showing exclusive nonsense mutations in the *X. perforans* T4 race from Taiwan (highlighted by the red square). Nonsense mutations are depicted in black. Polymorphic residues are depicted with colors. The race of each strain is identified within parentheses.

To characterize the effector composition of the Taiwanese population, we compared the Xanthomonas outer proteins (Xop) repertoires of 317 genomes and found evidence of diversification and local adaptation in Taiwan. Out of 70 Xops reported in the global database,^3^ only 48 were present in the target genomes (Supplementary Table S3). Similar to previous studies, we found a variable number of effectors in each strain (Schwartz et al., 2015; Roach et al., 2019), where *X. perforans* displayed 23 to 31 Xops while *X. euvesicatoria* displayed 30 to 31 Xops. We only found 11 Xop effectors in all the *X. vesicatoria* strains (Supplementary Table S3). Based on the presence/absence of effectors, the local *X. perforans*, *X. euvesicatoria*, and *X. vesicatoria* strains showed a unique composition compared to the global population (Figure 4A; Supplementary Figure S2). We identified at least 13 effector profiles in a sample of 21 genomes, with most of the unique profiles occurring in *X. perforans* genomes (8) compared to *X. euvesicatoria* genomes (4). The observed pattern is unlikely to result from an assembly or annotation issue since all the *X. vesicatoria* genomes assembled the same Xop genes and formed a single profile. The diversity in effector composition observed in *X. perforans* (Figure 4) aligns with the occurrence of multiple virulence patterns within T4 and T3 strains (Figure 2). Diversification of virulence factors has been reported in other pathogens and is likely the result of local adaptation (Quibod et al., 2016; Richards et al., 2019).

The comparative analysis of the Xops dataset also identified 13 effectors with rare alleles only present in Taiwan (Supplementary Table S4). These variations are characterized by one or more nonsynonymous mutations (Supplementary Table S4), which are most likely the result of adaptation to the local environment. Interestingly, we found most of the rare alleles in *X. perforans* genomes (Figure 4A). For instance, the strain Xant319 showed a unique amino acid substitution (S741G) in the highly conserved region of the protein xopZ1. We found derived alleles of xopD and xopV in *X. perforans* strains associated with recent collections (Supplementary Table S4), which might suggest more recent mutations. All of these genes have been reported to contribute to *Xanthomonas* colonization in various host systems (Furutani et al., 2009; Medina et al., 2018; Deb et al., 2020), and therefore, the observed mutations might have a fitness role in the local environment. In addition, strains Xant314 and Xant328 contain the effector *xopAZ*, which is absent from any of the genomes analyzed but was first reported in *X. arboricola* and *X. cynarae* (Kara et al., 2017). A few effectors also showed Indels and frameshift mutations that potentially disrupt their function (Supplementary Table S5). One clear example represents mutations in *xopAF/avrXv3* associated with race T4. Loss of avirulence function in this gene has been associated with a range of changes in the protein sequence (Timilsina et al., 2016; Bernal et al., 2022). We found two nonsense mutations in *xopAF* alleles from *X. perforans* T4 strains from Taiwan (Figure 4B). These mutations appear to be the only difference between *xopAF* alleles from race T3 and T4, suggesting that T4 strains emerged from T3 genetic backgrounds by random mutation (Bernal et al., 2022).

Interestingly, the effector repertoires of *X. vesicatoria* strains consist of only 11 genes (Figure 4A; Supplementary Table S3), which is shorter than any of the global *X. vesicatoria* genomes reported so far. The pathogenicity capabilities of these *X. vesicatoria* strains with a minimal number of effectors suggest a high plasticity of the pathogen associated with the redundancy of effector functions (Castaneda et al., 2005; Long et al., 2018). The overall data indicates that the genetic background of the *Xanthomonas* population from Taiwan appears to be narrow and most likely the result of a few colonization events that caused profound changes in population structure. However, the phenotypic pattern and the effector composition suggest that such a genetically uniform population is diversifying due to local adaptation.

## Conclusion

The introduction of *Xanthomonas* pathogens into Taiwan has led to significant shifts in disease dynamics across tomato-growing areas, particularly evident in the rapid dominance of *X. perforans* race T4. Despite that only a few pathogen genotypes managed to colonize the island, the population quickly diversified in response to local adaptation. These findings emphasize the complexity of managing introduced plant pathogens in semi-isolated environments and underscore the importance of ongoing research for effective disease responses.

## Data availability statement

The datasets presented in this study can be found in online repositories. The names of the repository/repositories and accession number(s) can be found in the article/Supplementary material.

## Author contributions

J-RC: Data curation, Formal analysis, Funding acquisition, Investigation, Methodology, Supervision, Validation, Writing – review & editing. KA-C: Data curation, Formal analysis, Investigation, Methodology, Software, Supervision, Validation, Writing – review & editing. D-YX: Data curation, Resources, Validation, Writing – review & editing. H-CC: Data curation, Formal analysis, Investigation, Methodology, Resources, Validation, Writing – review & editing. LA-M: Conceptualization, Data curation, Formal analysis, Validation, Writing – review & editing. Y-PL: Formal analysis, Software, Writing – review & editing. VA-J: Conceptualization, Data curation, Formal analysis, Investigation, Methodology, Project administration, Software, Supervision, Writing – original draft, Writing – review & editing. RO: Conceptualization, Funding acquisition, Resources, Supervision, Writing – original draft, Writing – review & editing.

## Footnotes

^1^ www.geneious.com

^2^ https://euroxanth.ipn.pt/doku.php?id=bacteria:t3e:t3e

^3^ https://euroxanth.eu/
