# Supplementary material for: Exploring the genetic makeup of Xanthomonas species causing bacterial spot in Taiwan: evidence of population shift and local adaptation
Source: Front Microbiol. 2024 May 23;15:1408885. doi: 10.3389/fmicb.2024.1408885 (PMC11153759; doi:10.3389/fmicb.2024.1408885)
Supplement: Supplementary file 2 [file Data_Sheet_1.PDF]

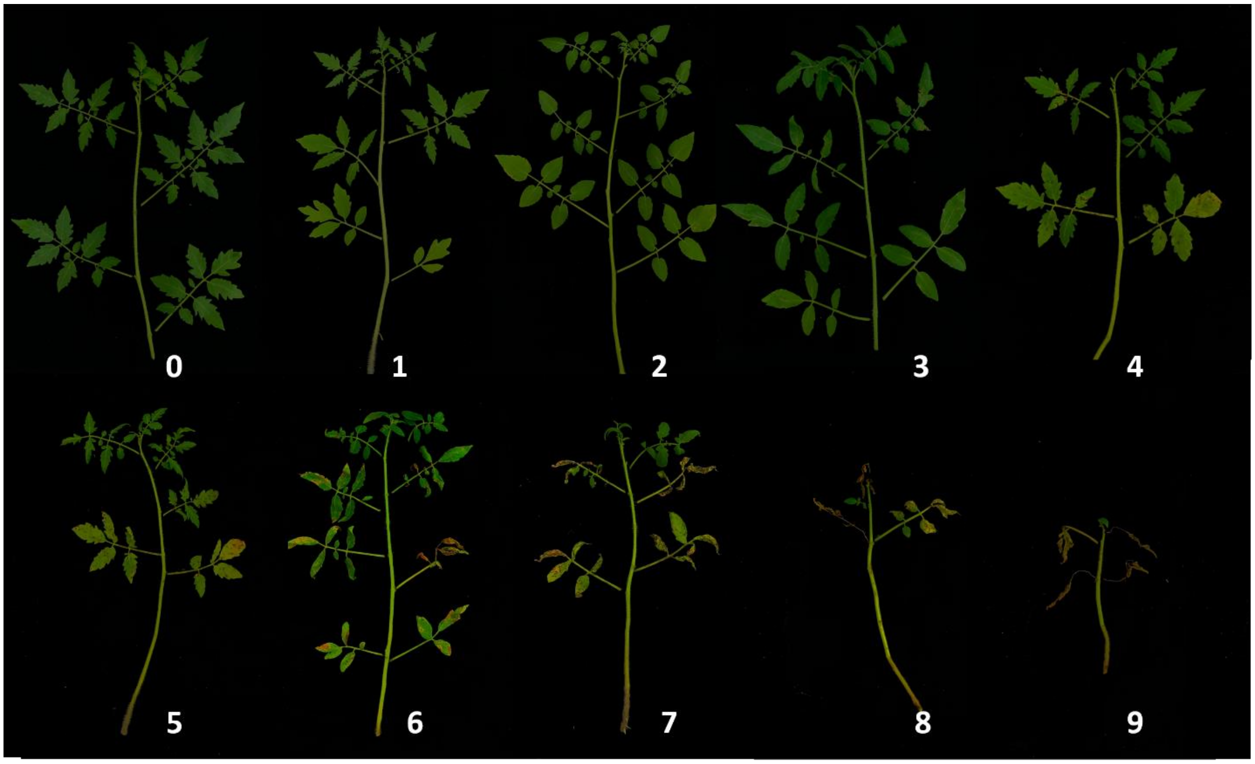

Figure S1. Disease scoring of tomato bacterial spot from 0-11. Scores from 1 to 3: the symptoms seemed to be no significant difference from the healthy plant and the spots often appear on single leaf. Scores of 4 to 5: the spots become obvious and the disease leaves turn chlorotic. Scores of 6 to 7: the spots fuse into large areas and seem to blight symptoms. Scores of 8 to 9: most leaves fall and the plants nearly die. Scores of 10 to 11: dead plants (not shown in the figure)
