# Supplementary material for: Exploring the genetic makeup of Xanthomonas species causing bacterial spot in Taiwan: evidence of population shift and local adaptation
Source: Front Microbiol. 2024 May 23;15:1408885. doi: 10.3389/fmicb.2024.1408885 (PMC11153759; doi:10.3389/fmicb.2024.1408885)
Supplement: Supplementary file 3 [file Data_Sheet_2.PDF]

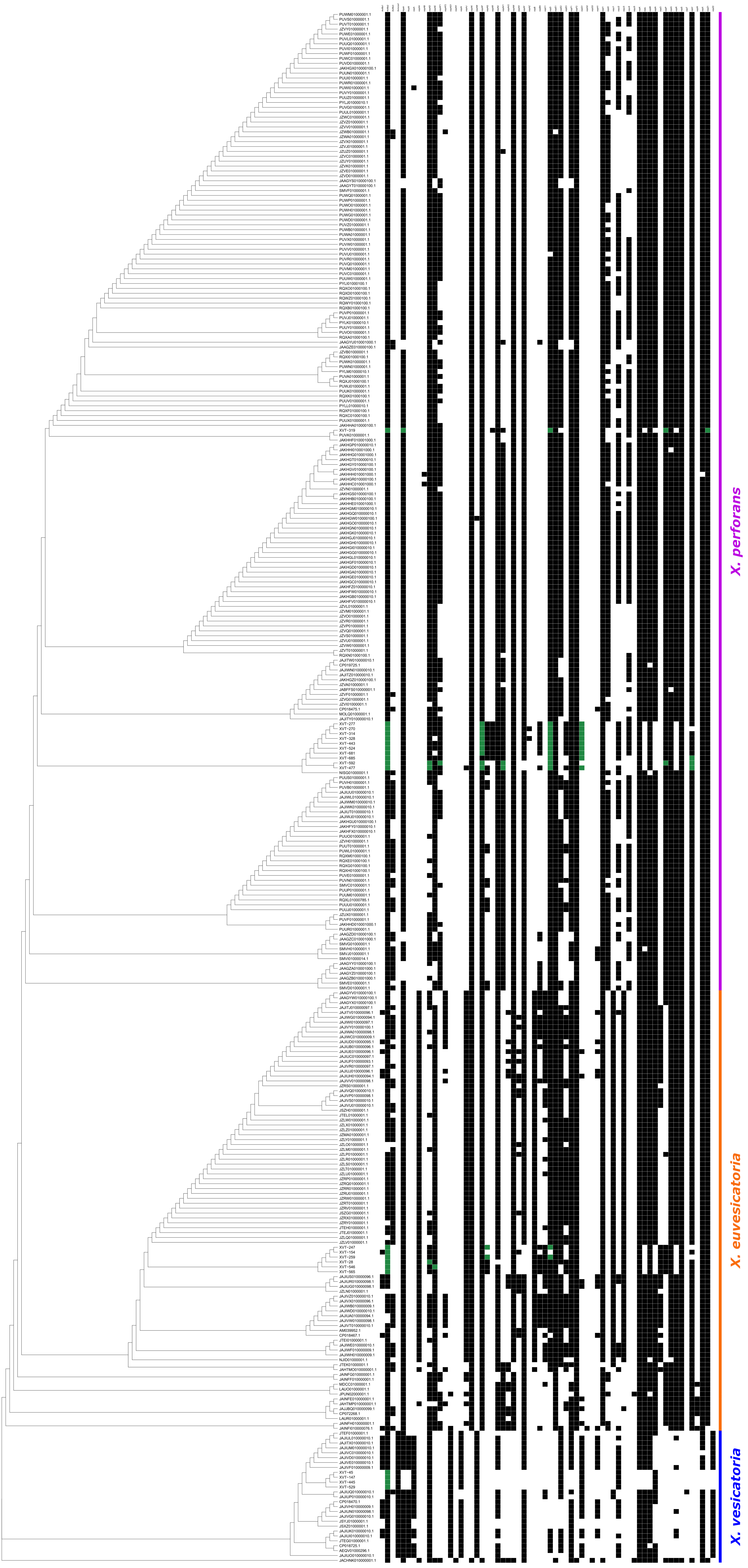

Figure S2. Phylogenetic reconstruction of 317 *Xanthomonas* genomes causing bacterial spot and a heatmap distribution of effectors genes. The dendrogram represents the phylogenetic relationships among *Xanthomonas vesicatoria*, *X. euvesicatoria*, and *X. perforans* strains. The heatmap grid shows the presence (black) or absence (white) of 64 known effector genes across multiple strains. Unique effectors are highlighted in green squares, showcasing the distinctiveness of certain strains coming from Taiwan.
